# Supplementary material for: A Systematic Review and a Meta-Analysis of the Yellow Fever Vaccine in the Elderly Population
Source: Vaccines (Basel). 2022 Apr 30;10(5):711. doi: 10.3390/vaccines10050711 (PMC9147422; doi:10.3390/vaccines10050711)

Supporting information

Table S1: Prisma checklist

Section and Topic	Item #	Checklist item	Location where item is reported (page)
**TITLE**			
Title	1	Identify the report as a systematic review.	1
**ABSTRACT**			
Abstract	2	See the PRISMA 2020 for Abstracts checklist.	2
**INTRODUCTION**			
Rationale	3	Describe the rationale for the review in the context of existing knowledge.	2-4
Objectives	4	Provide an explicit statement of the objective(s) or question(s) the review addresses.	4
**METHODS**			
Eligibility criteria	5	Specify the inclusion and exclusion criteria for the review and how studies were grouped for the syntheses.	5
Information sources	6	Specify all databases, registers, websites, organisations, reference lists and other sources searched or consulted to identify studies. Specify the date when each source was last searched or consulted.	4-5
Search strategy	7	Present the full search strategies for all databases, registers and websites, including any filters and limits used.	Supplementary table 2
Selection process	8	Specify the methods used to decide whether a study met the inclusion criteria of the review, including how many reviewers screened each record and each report retrieved, whether they worked independently, and if applicable, details of automation tools used in the process.	5
Data collection process	9	Specify the methods used to collect data from reports, including how many reviewers collected data from each report, whether they worked independently, any processes for obtaining or confirming data from study investigators, and if applicable, details of automation tools used in the process.	6-7
Data items	10a	List and define all outcomes for which data were sought. Specify whether all results that were compatible with each outcome domain in each study were sought (e.g. for all measures, time points, analyses), and if not, the methods used to	6-7

Section and Topic	Item #	Checklist item	Location where item is reported (page)
		decide which results to collect.	
	10b	List and define all other variables for which data were sought (e.g. participant and intervention characteristics, funding sources). Describe any assumptions made about any missing or unclear information.	6-7
Study risk of bias assessment	11	Specify the methods used to assess risk of bias in the included studies, including details of the tool(s) used, how many reviewers assessed each study and whether they worked independently, and if applicable, details of automation tools used in the process.	6
Effect measures	12	Specify for each outcome the effect measure(s) (e.g. risk ratio, mean difference) used in the synthesis or presentation of results.	6-7
Synthesis methods	13a	Describe the processes used to decide which studies were eligible for each synthesis (e.g. tabulating the study intervention characteristics and comparing against the planned groups for each synthesis (item #5)).	5
	13b	Describe any methods required to prepare the data for presentation or synthesis, such as handling of missing summary statistics, or data conversions.	5-7
	13c	Describe any methods used to tabulate or visually display results of individual studies and syntheses.	6-7
	13d	Describe any methods used to synthesize results and provide a rationale for the choice(s). If meta-analysis was performed, describe the model(s), method(s) to identify the presence and extent of statistical heterogeneity, and software package(s) used.	6-7
	13e	Describe any methods used to explore possible causes of heterogeneity among study results (e.g. subgroup analysis, meta-regression).	6-7
	13f	Describe any sensitivity analyses conducted to assess robustness of the synthesized results.	6-7
Reporting bias assessment	14	Describe any methods used to assess risk of bias due to missing results in a synthesis (arising from reporting biases).	5-7
Certainty assessment	15	Describe any methods used to assess certainty (or confidence) in the body of evidence for an outcome.	5
**RESULTS**			
Study selection	16a	Describe the results of the search and selection process, from the number of records identified in the search to the number of studies included in the review, ideally using a flow diagram.	7, F1
	16b	Cite studies that might appear to meet the inclusion criteria, but which were excluded, and explain why they were	7, F1

Section and Topic	Item #	Checklist item	Location where item is reported (page)
		excluded.	
Study characteristics	17	Cite each included study and present its characteristics.	7-11
Risk of bias in studies	18	Present assessments of risk of bias for each included study.	14
Results of individual studies	19	For all outcomes, present, for each study: (a) summary statistics for each group (where appropriate) and (b) an effect estimate and its precision (e.g. confidence/credible interval), ideally using structured tables or plots.	7-14
Results of syntheses	20a	For each synthesis, briefly summarise the characteristics and risk of bias among contributing studies.	14
	20b	Present results of all statistical syntheses conducted. If meta-analysis was done, present for each the summary estimate and its precision (e.g. confidence/credible interval) and measures of statistical heterogeneity. If comparing groups, describe the direction of the effect.	11-14
	20c	Present results of all investigations of possible causes of heterogeneity among study results.	11-14
	20d	Present results of all sensitivity analyses conducted to assess the robustness of the synthesized results.	11-14
Reporting biases	21	Present assessments of risk of bias due to missing results (arising from reporting biases) for each synthesis assessed.	11-14
Certainty of evidence	22	Present assessments of certainty (or confidence) in the body of evidence for each outcome assessed.	11-14
**DISCUSSION**			
Discussion	23a	Provide a general interpretation of the results in the context of other evidence.	14-18
	23b	Discuss any limitations of the evidence included in the review.	16-17
	23c	Discuss any limitations of the review processes used.	16-17
	23d	Discuss implications of the results for practice, policy, and future research.	17-18
**OTHER INFORMATION**			
Registration and protocol	24a	Provide registration information for the review, including register name and registration number, or state that the review was not registered.	4

Section and Topic	Item #	Checklist item	Location where item is reported (page)
	24b	Indicate where the review protocol can be accessed, or state that a protocol was not prepared.	4
	24c	Describe and explain any amendments to information provided at registration or in the protocol.	4
Support	25	Describe sources of financial or non-financial support for the review, and the role of the funders or sponsors in the review.	19
Competing interests	26	Declare any competing interests of review authors.	19
Availability of data, code and other materials	27	Report which of the following are publicly available and where they can be found: template data collection forms; data extracted from included studies; data used for all analyses; analytic code; any other materials used in the review.	4

Table S2: List of search terms per database assessed for the review in September 2020 and updated in December 2021.

Database	Search strategy
Medline	(((((((((((((((((((aged[MeSH Terms]) OR aged[Title/Abstract]) OR (aged, 80 and over[MeSH Terms])) OR (aged, 80[Title/Abstract] AND over[Title/Abstract])) OR elder[MeSH Terms]) OR elder[Title/Abstract]) OR elders[MeSH Terms]) OR elders[Title/Abstract]) OR geriatric[MeSH Terms]) OR geriatric[Title/Abstract]) OR old people[MeSH Terms]) OR old people[Title/Abstract]) OR senior[MeSH Terms]) OR senior[Title/Abstract]) OR elderly[MeSH Terms]) OR elderly[Title/Abstract]) OR aging[MeSH Terms]) OR aging[Title/Abstract])) AND ((((((((((("yellow fever vaccine"[Title/Abstract]) OR Yellow Fever Vaccine[MeSH Terms]) OR vaccine, yellow fever[Title/Abstract]) OR vaccine, yellow fever[MeSH Terms]) OR yellow fever vaccination[Title/Abstract]) OR yellow fever vaccination[MeSH Terms]) OR 17D vaccin*[Title/Abstract]) OR 17D-204 vaccin*[Title/Abstract]) OR 17DD vaccin*[Title/Abstract]) OR asibi strain*[Title/Abstract]) OR YF-VAX[Title/Abstract])) AND ((adverse effects[Title/Abstract]) OR adverse effects[MeSH Terms]) OR adverse effects/complications[Title/Abstract]) OR adverse effects/complications[MeSH Terms]) OR adverse effects/side effects/safety[Title/Abstract]) OR adverse effects/side effects/safety[MeSH Terms]) OR interaction response[Title/Abstract]) OR interaction response[MeSH Terms]) OR adverse effect[Title/Abstract]) OR adverse effect[MeSH Terms]) OR adverse event[Title/Abstract]) OR reaction[Title/Abstract]) OR reaction[MeSH Terms]) OR side effect[Title/Abstract]) OR vaccination/adverse effects[Title/Abstract]) OR vaccination/adverse effects[MeSH Terms]) OR *adverse events following immunization[Title/Abstract]) OR vaccine surveillance[Title/Abstract]) OR vaccine surveillance[MeSH Terms]) OR postmarketing[Title/Abstract]) OR Product Surveillance, Postmarketing[MeSH Terms]) OR postmarketing adverse event[Title/Abstract]) OR harm[Title/Abstract]) OR harm[MeSH Terms]) OR vaccine safety[Title/Abstract]) OR vaccine safety[MeSH Terms]) OR safety[Title/Abstract]) OR safety[MeSH Terms]) OR vaccine effects[Title/Abstract]) OR vaccine effects[MeSH Terms]) OR specific adverse effects[Title/Abstract]) OR specific adverse effects[MeSH Terms]) OR risk[Title/Abstract]) OR risk[MeSH Terms]) OR guillain barre syndrome[Title/Abstract]) OR guillain barre syndrome[MeSH Terms]) OR Guillain-Barre Syndrome[Title/Abstract]) OR Guillain-Barre Syndrome[MeSH Terms]) OR encephalomyelitis[Title/Abstract]) OR encephalomyelitis[MeSH Terms]) OR acute disseminated encephalomyelitis[Title/Abstract]) OR acute disseminated encephalomyelitis[MeSH Terms]) OR yellow fever vaccine/adverse effects[Title/Abstract]) OR yellow fever vaccine/adverse effects[MeSH Terms]) OR meningoencephalitis[Title/Abstract]) OR meningoencephalitis[MeSH Terms]) OR vaccine associated neurologic disease[Title/Abstract]) OR vaccine associated neurologic disease[MeSH Terms]) OR

	nerotropic[Title/Abstract]) OR nerotropic[MeSH Terms]) OR nervous system diseases[Title/Abstract]) OR nervous system diseases[MeSH Terms]) OR vaccine associated viscerotropic disease[Title/Abstract]) OR vaccine associated viscerotropic disease[MeSH Terms]) OR viscerotropic[Title/Abstract]) OR viscerotropic[MeSH Terms]) OR viscerotropic adverse events[Title/Abstract]) OR viscerotropic adverse events[MeSH Terms]) OR *adverse drug reaction reporting system[Title/Abstract]) OR viscerotropic disease[Title/Abstract]) OR viscerotropic disease[MeSH Terms]) OR anaphylactic[Title/Abstract]) OR anaphylactic[MeSH Terms]) OR hypersensitivity[Title/Abstract]) OR hypersensitivity[MeSH Terms]) OR anaphylactic reactions[Title/Abstract]) OR anaphylactic reactions[MeSH Terms]) OR death[Title/Abstract]) OR death[MeSH Terms]) OR mortality[Title/Abstract]) OR mortality[MeSH Terms]) OR multiple organ failure[Title/Abstract]) OR multiple organ failure[MeSH Terms])
Embase	('very elderly'/exp OR 'aged'/exp OR older OR aging OR senior$ OR (old AND people) OR elderly OR geriatric$ OR elderes) AND ('yellow fever vaccine'/exp OR 'yf vax' OR stamaril OR 'yellow fever vaccine'/exp/dd_ae OR (17d AND vaccin*) OR ('17d 204' AND vaccin*) OR (17dd AND vaccin*) OR (asibi AND strain*)) AND ('adverse event'/exp OR 'vaccine'/exp/dd_ae OR 'side effect'/exp OR 'safety'/exp OR 'postmarketing surveillance'/exp OR 'guillain barre syndrome' OR encephalomyelitis OR 'acute disseminated encephalomyelitis' OR meningoencephalitis OR (vaccine AND associated AND neurologic AND disease) OR (vaccine AND associated AND neurotropic AND disease) OR neurotropic OR 'neurologic disease'/exp OR (vaccine AND associated AND viscerotropic AND disease) OR viscerotropic OR (viscerotropic AND adverse AND events) OR 'viscerotropic disease' OR 'anaphylaxis'/exp OR death OR mortality OR (multiple AND organ AND failure))
Scopus	((TITLE-ABS-KEY (aged) OR TITLE-ABS-KEY (elderly) OR TITLE-ABS-KEY (aged 80 AND over) OR TITLE-ABS-KEY (senior) OR TITLE-ABS-KEY (old) OR TITLE-ABS-KEY (old AND people) OR TITLE-ABS-KEY (aging) OR TITLE-ABS-KEY (geriatric))) AND (TITLE-ABS-KEY ("Yellow Fever Vaccine" OR "yellow fever vaccination" OR "Yellow Fever Vaccine" OR "17D vaccine" OR "17D-204 vaccine" OR "17DD vaccine" OR "asibi strain" OR "YF-VAX")) AND ((TITLE-ABS-KEY ("adverse effects" OR "complications" OR "side effects" OR "safety" OR "interaction response" OR "effect" OR "event" OR "reaction" OR "side effect" OR "vaccination adverse effects" OR "adverse events following immunization" OR "vaccine surveillance") OR TITLE-ABS-KEY ("postmarketing" OR "postmarketing adverse event" OR "harm" OR "vaccine safety" OR "vaccine effects" OR "specific adverse effects" OR "risk" OR "guillain barre syndrome" OR "encephalomyelitis" OR "acute disseminated encephalomyelitis") OR TITLE-ABS-KEY ("meningoencephalitis" OR "vaccine associated neurologic disease" OR "vaccine associated neurotropic disease" OR "nerotropic" OR "nervous system diseases" OR "vaccine associated viscerotropic disease" OR "viscerotropic") OR TITLE-ABS-KEY ("viscerotropic adverse events" OR "adverse drug reaction reporting system" OR "viscerotropic

	disease" OR "anaphylactic" OR "hypersensitivity" OR "anaphylactic reactions" OR "death" OR "mortality" OR "multiple organ failure")))
Lilacs	(tw:((mh:("aged")) OR (mh:(aged 80 and over)) OR (mh:("elderly")) OR (tw:(elderly)) OR (tw:(geriatric$)) OR (tw:(senior$)) OR (mh:("older adult")) OR (tw:(aging)) OR (tw:(old people)))) AND (tw:((mh:("yellow fever vaccine")) OR (mh:("yellow fever vaccine/ adverse effects")) OR (mh:("yellow fever vaccine/ toxicity")) OR (tw:("yellow fever vaccination")) OR (tw:(17D vaccin*)) OR (tw:(17DD vaccin*)) OR (tw:(17D-204 vaccin*)) OR (tw:(asibi strain*)) OR (tw:("yf-vax")))) AND (tw:((tw:("adverse effects")) OR (tw:("adverse event")) OR (tw:("side effect")) OR (mh:("Product Surveillance, Postmarketing")) OR (mh:("risk management")) OR (mh:("safety")) OR (tw:("yellow fever vaccine/toxicity")) OR (mh:("vaccine/toxicity")) OR (tw:("reaction")) OR (mh:("vaccination/ adverse effect")) OR (tw:("postmarketing adverse event")) OR (tw:("harm*")) OR (tw:("vaccine safety")) OR (tw:("vaccine effects")) OR (mh:("acute disseminated encephalomyelitis")) OR (mh:("meningoencephalitis")) OR (tw:("vaccine associated neurotropic disease")) OR (tw:("neurotropic")) OR (tw:("vaccine associated viscerotropic disease")) OR (tw:("viscerotropic")) OR (tw:("*adverse drug reaction reporting system")) OR (tw:("viscerotropic disease")) OR (mh:("Anaphylaxis")) OR (mh:("Hypersensitivity")) OR (mh:("death")) OR (mh:("yellow fever vaccine/mortality"))))
Toxiline	'aged AND yellow fever vaccine'
Database of Abstracts of Review of Effects	((aged) OR (elderly)) AND ((yellow fever vaccine))
Web of Science	TÓPICO: (("aged" OR "elderly" OR "aged 80 and over" OR "senior" OR "old" OR "old people" OR "aging" OR "geriatric")) *AND* TÓPICO: (("Yellow Fever Vaccine" OR "vaccine yellow fever" OR "yellow fever vaccination" OR "Yellow Fever Vaccine" OR 17D vaccin* OR 17D-204 vaccin* OR 17DD vaccin* OR asibi strain* OR "YF-VAX")) *AND* TÓPICO: (("adverse effects" OR "adverse effects/complications" OR "adverse effects/side effects/safety" OR "interaction response" OR "adverse effect" OR "adverse event" OR "reaction" OR "side effect" OR "vaccination/adverse effects" OR "*adverse events following immunization" OR "vaccine surveillance" OR "postmarketing" OR "Product Surveillance Postmarketing" OR "postmarketing adverse event" OR "harm" OR "vaccine safety" OR "safety" OR "vaccine effects" OR "specific adverse effects" OR "risk" OR "guillain barre syndrome" OR "Guillain-Barre Syndrome" OR "encephalomyelitis" OR "acute disseminated encephalomyelitis" OR "yellow fever vaccine/adverse effects" OR "meningoencephalitis" OR "vaccine associated neurologic disease" OR "nerotropic" OR "nervous system diseases" OR "vaccine associated viscerotropic disease" OR "viscerotropic" OR "viscerotropic adverse events" OR "*adverse drug reaction reporting system" OR "viscerotropic disease" OR "hypersensitivity" OR "anaphylactic reactions" OR "death" OR "mortality" OR "multiple organ failure"))
Open Grey	"Yellow fever vaccine AND Elderly"
Grey Literature Report	"Yellow fever vaccine AND Elderly"

CDC Morbidity and Mortality Weekly Report (MMWR)	"Yellow fever vaccine" AND "elderly"
Vaccine Adverse Event Reporting System (VAERS)	"Yellow fever vaccine"
Australian Adverse Drug Reactions Bulletin	"yellow fever vaccine" OR "stamaril"
European Database of Suspected Adverse Drug Reaction Reports (EudraVigilance)	"yellow fever vaccine" OR "stamaril"
FDA Adverse Event Reporting System (FAERS)	"yellow fever vaccine" OR "stamaril" OR "yf-vax"
Institutional Repository for Information Sharing - World Health Organization (IRIS)	"yellow fever vaccine"
11th São Paulo Congress of Infectology	"yellow fever vaccine"
ClinicalTrials.gov	"yellow fever vaccine" OR "Yellow fever"

Table S3: Total serious adverse events reported in studies not included for the meta-analysis.

**Study**	**Age group assessed**	**Number of SAE**	**Viscerotropic disease associated with YFV**	**Neurological manifestations associated with YFV**	**Deaths**
CDC, 2002[26]	≥70	2	1	1	0
Azevedo et al, 2011[27]	≥65	0	0	0	0
Bae et al, 2008[28]	≥61	3	3	0	0
Cottin et al, 2013[30]	≥60	11	6	5	0
Mota et al,2009[31]	≥60	0	0	0	0
Martin et al, 2001[38]	≥63	4	4	0	3
McMahon et al, 2006[39]	≥60	6	0	6	0
Lucena et al, 2020[41]	≥60	12	Missing information	Missing information	Missing information

Table S4: Result of meta-regression between characteristics of age, study design and type of serious adverse event.

	estimate	se	zval	pval	ci.lb	ci.ub	
intrcpt	2.0878	0.5000	4.1757	<0.0001	1.1078	3.0677	***
age_group <70 years	-1.0706	0.4778	-2.2407	0.0250	-2.0072	-0.1341	*
age_group elderly with no age specification	-1.5723	0.6010	-2.6159	0.0089	-2.7503	-0.3942	**
study_design non-longitudinal	-0.4960	0.3988	-1.2436	0.2136	-1.2776	0.2857	
event_type without specification	0.7739	0.6152	1.2581	0.2083	-0.4317	1.9796	

Reference for p value: * 0.05. ** 0.01. *** 0.001.

Table S5. Characteristics of the interventions evaluated in the studies included in the review.

Characteristics	n	%
Vaccine lineage		
17D	7	38.9
17DD	3	16.7
17D-204	0	0.0
More than one lineage assessed	1	5.6
Missing information	7	38.9
Type of dose administrated		
Primary	2	11.1
Booster	0	0.0
Missing information	16	88.9
Vaccination type		
Campaign	1	5.6
Routine	2	11.1
Traveller	4	22.5
Missing information	11	61.1
Coadministration		
No	1	5.6
Yes	8	44.4
Missing information	9	50.0
Vaccine coadministration		
Typhoid fever	6	18.2
Hepatitis A	8	24.2
Hepatitis B	3	9.1
Meningococcal	2	6.1
Polio	4	12.1
Missing information	10	30.1

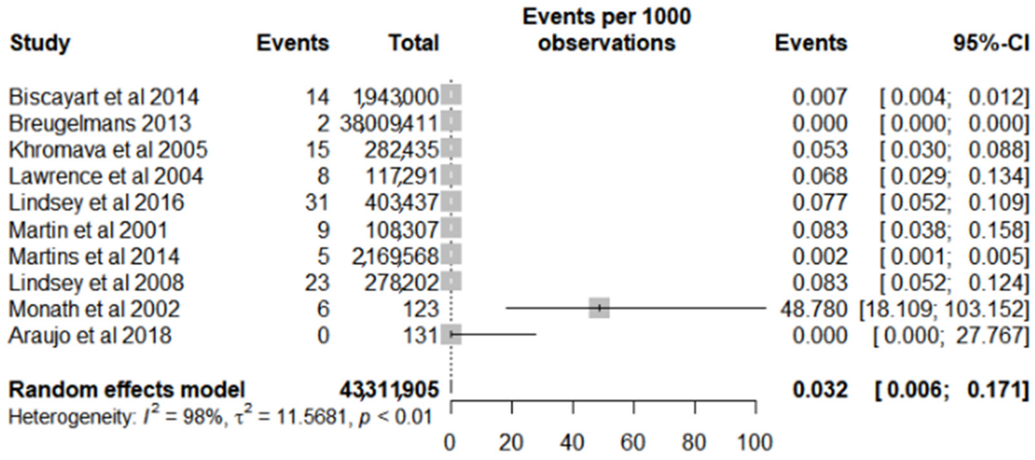

Figure S1: Forest plot of prevalence meta-analysis for serious adverse events after yellow fever vaccine use in the elderly.

Figure S2. Risk of bias assessment results according to domain and study included in the systematic review for the ROBINS-I tool.

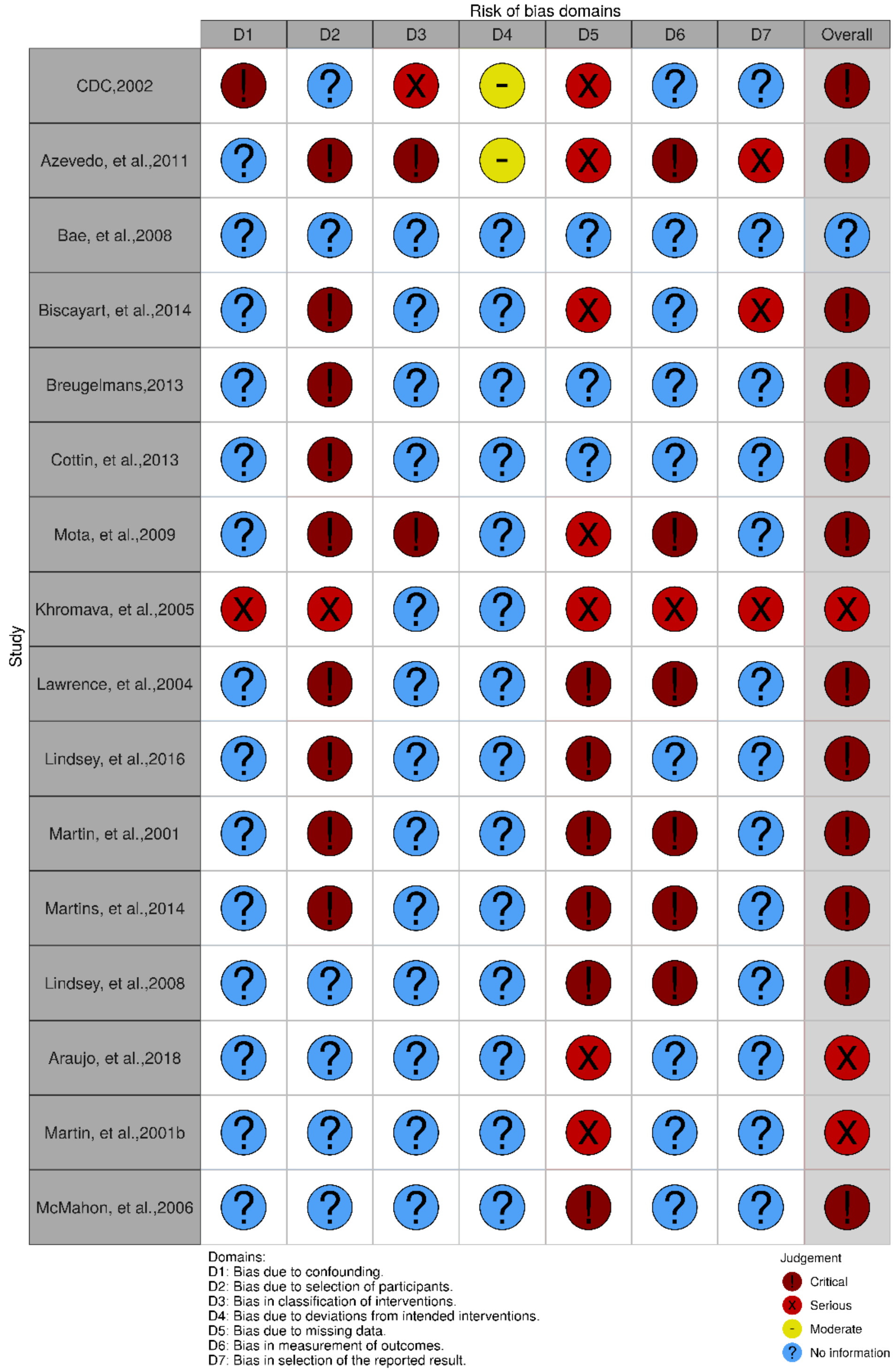

Figure S3. Risk of assessment results according to domain and study included in the systematic review for the RoB 2.0 tool.

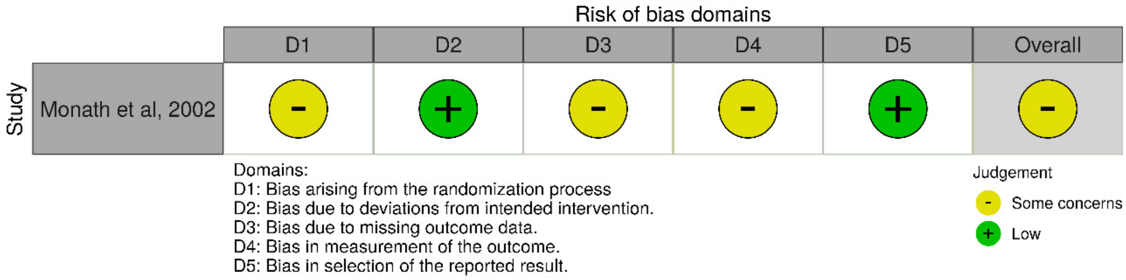

Supplement: Supplementary file 1 [file vaccines-10-00711-s001.zip › vaccines-1698966-supplementary.pdf]
